# Enhancing drought resistance in *Pinus tabuliformis* seedlings through root symbiotic fungi inoculation

**DOI:** 10.3389/fpls.2024.1446437

**Published:** 2024-08-20

**Authors:** Lingjie Xu, Jiadong He, Yu Meng, Yanyan Zheng, Bin Lu, Jiawen Zhang, Yong Zhou

**Affiliations:** ^1^ Country College of Landscape Architecture and Tourism, Hebei Agricultural University, Baoding, China; ^2^ Earth and Life Institute, Université catholique de Louvain-UCLouvain, Louvain-la-Neuve, Belgium

**Keywords:** antioxidant activities, ectomycorrhizal fungi, mixed inoculation, osmotic adjustment, PEG-6000, root symbiotic fungi

## Abstract

**Background:**

Drought constitutes a major abiotic stress factor adversely affecting plant growth and productivity. Plant-microbe symbiotic associations have evolved regulatory mechanisms to adapt to environmental stress conditions. However, the interactive effects of different fungi on host growth and stress tolerance under drought conditions remain unclear.

**Objective:**

This study explored the effects of varying polyethylene glycol (PEG-6000) concentrations (0%, 15%, 25%, and 35%) on the growth and physiological responses of two ectomycorrhizal fungi (*Suillus granulatus* (Sg) and *Pisolithus tinctorius* (Pt)) and two dark septate endophytes (*Pleotrichocladium opacum* (Po) and *Pseudopyrenochaeta* sp. (Ps)) isolated from the root system of *Pinus tabuliformis*. Specifically, the study aimed to evaluate six inoculation treatments, including no inoculation (CK), single inoculations with Sg, Pt, Po, Ps, and a mixed inoculation (Sg: Pt : Po: Ps = 1:1:1:1), on the growth and physiological characteristics of *P. tabuliformis* seedlings under different water regimes: well-watered at 70% ± 5%, light drought at 50% ± 5%, and severe drought at 30% ± 5% of the maximum field water holding capacity.

**Results:**

All four fungi exhibited the capacity to cope with drought stress by enhancing antioxidant activities and regulating osmotic balance. Upon successful root colonization, they increased plant height, shoot biomass, root biomass, total biomass, and mycorrhizal growth response in *P. tabuliformis* seedlings. Under drought stress conditions, fungal inoculation improved seedling drought resistance by increasing superoxide dismutase and catalase activities, free proline and soluble protein contents, and promoting nitrogen and phosphorus uptake. Notably, mixed inoculation treatments significantly enhanced antioxidant capacity, osmotic adjustment, and nutrient acquisition abilities, leading to superior growth promotion effects under drought stress compared to single inoculation treatments.

**Conclusion:**

All four fungi tolerated PEG-induced drought stress, with increased antioxidant enzyme activities and osmotic adjustment substances and they promoted the growth and enhanced drought resistance of *P. tabuliformis* seedlings.

## 1 Introduction

Currently, the trend of global climate change is characterized by rising temperatures and increasing aridity, with precipitation patterns undergoing noticeable changes. The frequency, duration, intensity, and area of droughts have increased significantly. Drought has emerged as a principal factor impacting vegetation growth and recovery (Cook et al., 2014; Huang et al., 2016; Kumar et al., 2024). In arid regions, high summer temperatures adversely affect the survival of tree seedlings and hinder forest regeneration. Simultaneously, soil temperature indirectly influences nutrient synthesis, translocation, and absorption, thereby disrupting the normal physiological processes of young seedlings and eventually impeding vegetation growth and regeneration within the entire forest ecosystem (Chidumayo, 2008). Although drought diminishes soil productivity, constrains water uptake, induces plant osmotic and redox imbalances, and ultimately leads to plant mortality (Alori et al., 2020; Farrell et al., 2017), microbial interventions can effectively alleviate drought stress (Saritha et al., 2021; Li and Liu, 2016; Salehi-Lisar and Bakhshayeshan-Agdam, 2016). Studies have demonstrated that when plants establish mycorrhizal associations in their root systems, their capacity to absorb water and nutrients significantly improves, thereby enhancing their drought resistance (Gleason et al., 2006). Consequently, the inoculation of beneficial fungi represents an effective strategy for maintaining plant health and quality, necessitating the selection of fungal species capable of adapting to drought conditions and the screening of strains and strain combinations exhibiting robust drought tolerance.

Ectomycorrhizal fungi (ECMF) establish ectomycorrhizal associations when their mycelia infect the nutrient roots of host plants. The primary host plants of ECMF belong to the Betulaceae, Pinaceae, Salicaceae, and Fagaceae families (Tedersoo and Brundrett, 2017). Existing studies indicate that Pinaceae are among the earliest ectomycorrhizal plant lineages (Tedersoo et al., 2012). Dark septate endophytes (DSE) are a type of minute endophytes that colonize the epidermal cells, root epidermis, cortex, and even the vascular tissue cells or intercellular spaces of plant roots, forming dark-colored, conspicuous structures with laterally septate hyphae and microsclerotia (Addy et al., 2005; Mandyam et al., 2010; Gaber et al., 2023). These fungi exhibit a high colonization rate in plant roots under drought or extreme environmental conditions (Gucwa-Przepióra et al., 2016), with a broad host range encompassing nearly 600 plant species from 320 genera across 114 families (Jumpponen et al., 1998), garnering increasing research attention in recent years (Santos et al., 2021; Huertas et al., 2024). Both ECMF and DSE promote plant growth and improve stress tolerance, playing crucial roles in ecosystem stability and restoration (Yin et al., 2018; Li et al., 2022a, b). Recent studies by Zhang et al. (2024) revealed that inoculating ECMF strains enhanced the drought resistance of *Pinus massoniana* seedlings during the early stages of drought stress by influencing water content, photosynthesis, osmolyte accumulation, and antioxidant enzyme activities in the shoots and roots. Similarly, Wang et al. (2021) reported that ECMF inoculation increased water content, photosynthetic rate, and osmolyte accumulation in *P. tabuliformis* under drought stress. Li et al. (2021) demonstrated that DSE inoculation could increase the biomass of *Isatis indigotica* and mitigate oxidative damage caused by drought stress. However, the interactive effects of these two fungi on host growth and stress tolerance under drought conditions remain unclear, with limited research exploring the co-existence of both fungi and their impact on host plant drought tolerance.

In recent years, the study of co-inoculation with ectomycorrhizal fungi has emerged as a prominent topic in rhizosphere microecology research (Corrales et al., 2022; Tedersoo et al., 2024). The combined application of ectomycorrhizal fungi and Trichoderma has been shown to effectively enhance the antioxidant capacity of Scots pine seedlings (Yin et al., 2016). Deng et al. (2017) reported that dual inoculation with *Suillus luteus* and dark septate endophytes improved the resistance of *Pinus sylvestris* var. *mongolica* seedlings to damping-off disease, reduced seedling blight incidence, and increased seedling survival rates. Our previous studies have demonstrated that inoculating two ECMF and two DSE strains could improve the growth, root development, nutrient uptake, and soil microbial community composition of *P. tabuliformis* seedlings (Xu et al., 2022), and effectively regulate the antioxidant defense response and photosynthesis of these seedlings under cadmium stress. The co-inoculation of the two fungi exhibited a more pronounced synergistic effect on plant tolerance to heavy metals (Zhou et al., 2024). Although the direct effects of beneficial microbial inoculants on plant growth have been widely reported (Santos et al., 2017; He et al., 2019), information regarding the contribution of the ECMF and DSE combination to plant growth under drought stress remains limited.

*Pinus tabuliformis*, the predominant tree species in North China, holds immense importance for soil and water conservation, landscape aesthetics, and ecological balance within its distribution range (Gao et al., 2023). *P. tabuliformis* exhibits sensitivity to climate change-induced warming and drying. North China represents the most suitable growth area and concentrated distribution region for *P. tabuliformis*. The recruitment and renewal of *P. tabuliformis* seedlings profoundly influence the structure and species composition of forest communities, bearing great significance for forest resource reserves in this region. The ecological stability of China’s northern regions is facing a more serious threat in the face of increasingly severe drought. As the main afforestation species of plantation forests in the northern region, the plantation forests of *P. tabuliformis*, under the influence of prolonged and frequent droughts, have suffered from degradation of forest stands, poor natural regeneration, drying up of tree tops and sparse understory vegetation, which have led to a vicious cycle of ecological environment in the region and further accelerated the depletion of forest resources (Chen et al., 2015). Previous studies have revealed the widespread presence of both ECMF and DSE in the roots of *P. tabuliformis* (Chu et al., 2016, 2019, 2021; Xu et al., 2022).

Therefore, in this study, drought tolerance investigations were conducted on isolated, purified, and identified root symbiotic fungi of *P. tabuliformis* to screen for strains exhibiting robust drought resistance. Subsequently, the root symbiotic fungi were reinoculated into *P. tabuliformis* seedlings, and water control experiments were carried out in a greenhouse. This research aims to provide a reference for further investigation of the drought resistance mechanisms of root symbiotic fungi and the screening of drought-resistant fungi. It also offers theoretical and technical support for *P. tabuliformis* afforestation in arid areas, promoting understory regeneration, preventing soil erosion, and improving forest resource quality. Previous studies on the resistance of mycorrhizal fungi in plants have predominantly focused on single inoculation methods. In contrast, this study introduces a mixed inoculation approach to evaluate the effects of different inoculation methods on plant-microbe symbionts. This experiment primarily examines the physiological and biochemical responses of these symbionts under drought stress conditions. While current research has provided valuable insights, the molecular mechanisms by which fungi enhance drought tolerance in *P. tabuliformis* remain underexplored. Future research should leverage advanced molecular biology techniques, such as transcriptomics, proteomics, and metabolomics, to elucidate these mechanisms.

## 2 Materials and methods

### 2.1 Biological material

The four-plant root symbiotic fungi used in this experiment were two ECMF, *Suillus granulatus* (*Sg*) and *Pisolithus tinctorius* (*Pt*), and two DSE, *Pleotrichocladium opacum* (*Po*) and *Pseudopyrenochaeta* sp. (*Ps*), which were isolated from the root of *P. tabuliformis* (Xu et al., 2022). These fungi were identified by morphological characteristics and phylogenetic analysis of nuclear ribosomal DNA (nrDNA) internal transcribed spacer (ITS) sequences (**Supplementary Figures S1**, **S2**). The fungi were cultured on potato dextrose agar media and stored in a 27°C mould incubator.

Uniformly sized and plump fresh seeds of *P. tabuliformis* were used in the experiments. The seeds were surface-sterilized and placed in Petri dishes in a 25°C light incubator to accelerate germination. Germinated seeds were sown in transparent plastic pots (15 cm height, 8 cm bottom diameter, 12 cm top diameter) filled with sterilized river sand (2 mm sieve, 121°C, 0.1 MPa, 2 h autoclaving). Each pot contained 1,000 g of the sterile sand medium, with four seeds sown per pot. During the seedling cultivation period, sterile water and Hoagland nutrient solution were supplied to ensure normal seedling growth.

### 2.2 Design of the experiment

#### 2.2.1 *In vitro* drought stress tolerance assay

The drought stress tolerance of four root symbiotic fungi (*Sg*, *Pt*, *Po* and *Ps*) isolated from *P. tabuliformis* was evaluated through solid and liquid culture experiments using polyethylene glycol (PEG-6000) to simulate drought stress. Media containing 0%, 15%, 25%, and 35% PEG-6000 were prepared using potato, glucose, plant gel, and PEG-6000. The experiment comprised 16 treatments with five replicates per treatment. Well-growing fungal colonies on potato dextrose agar were selected, and 6 mm fungal disks were inoculated into 20 mL solid media and 100 mL liquid media, respectively. Solid cultures were incubated at 27°C for 14 days, during which fungal colony morphology was observed and recorded. Liquid cultures were incubated in a thermostatic incubator at 27°C, with continuous shaking at 150 rpm, for 14 days in the dark. After this period, the mycelia were harvested to determine biomass and physiological indices.

#### 2.2.2 Pot experiment

A greenhouse pot experiment was conducted with different fungal inoculation treatments and moisture levels as variables. The experiment was performed under natural light conditions at Hebei Agricultural University, with temperatures maintained at 30°C during the day and 21°C at night, a 14-h light/10-h dark photoperiod, and relative humidity of 60% during the day and 70% at night. Six inoculation treatments were employed: no inoculation (CK), single inoculations with *Sg*, *Pt*, *Po*, or *Ps*, and a mixed inoculation (*Sg: Pt : Po: Ps* = 1:1:1:1). Liquid inocula was prepared by inoculating three fungal disks of each fungus into 150 mL potato dextrose broth and incubating on a shaking table at 27°C for 14 days. The mycelia were then homogenized and mixed with sterile water (v:v = 3:1) to obtain a mycelial suspension. Seedlings were inoculated by perforated root irrigation with 20 mL of the respective liquid inoculum, while the control group received 20 mL of potato dextrose broth.

One month post-inoculation, root samples were examined to confirm fungal colonization. Drought stress treatments were then imposed, with three soil moisture levels: 70% ± 5% (well-watered, WW), 50% ± 5% (light drought, LD), and 30% ± 5% (severe drought, SD) of the maximum field moisture capacity. Unified watering management was implemented, maintaining soil moisture content within the experimental range by weighing the pots at 17:00 every day. Plastic pots were repositioned every two weeks to minimize positional effects. The experiment consisted of 18 treatments with five replications each. After 40 days of drought stress, seedlings were harvested for relevant index measurements.

### 2.3 Antioxidant enzyme activity determination

The superoxide dismutase (SOD) activity in fungi and plants was determined by the nitroblue tetrazolium photoreduction method (Elavarthi and Martin, 2010), and the catalase (CAT) activity was assessed by the ultraviolet absorption method (Zhang, 1990).

### 2.4 Malondialdehyde and osmolyte determination

The malondialdehyde (MDA) content was quantified using the thiobarbituric acid method (Zhang, 1990), and proline content was determined following the method of Bates et al. (1973). Soluble protein content was measured by the Coomassie brilliant blue colorimetric method (Campion et al., 2017).

### 2.5 Fungal colonization rate determination

The ectomycorrhizal root colonization rates were calculated by microscopic examination of mycorrhizal and non-mycorrhizal root tips, and the percentages of root tips with evident ectomycorrhizal structures were determined (Brundrett et al., 1996). Dark septate endophyte colonization rates were assessed by trypan blue staining and microscopic quantification of septate hyphae and microsclerotia, with the percentage of colonized root segments calculated (Phillips and Hayman, 1970).

### 2.6 Mycorrhizal growth response calculation

The mycorrhizal growth response (MGR) was calculated according to the method of van der Heijden (2002). If the total dry weight of inoculated plants (M) exceeded the mean total dry weight of non-inoculated plants (NMmean), then MGR (%) = 100 × (1 − NMmean/M). If M < NMmean, then MGR (%) = 100 × (−1 + M/NMmean). A positive MGR value indicated that the inoculation treatment promoted plant growth, while a negative value signified growth inhibition.

### 2.7 Plant nutrient element analysis

The dried above-ground and below-ground portions of *P. tabuliformis* seedlings were ground into a fine powder and digested using the H_2_SO_4_-H_2_O_2_ method until the digestion solution became colorless and transparent (Bao, 2000). The digested solution was filtered and used to determine nitrogen and phosphorus contents in the aboveground and belowground parts by the Kjeldahl method and molybdenum antimony colorimetric method, respectively.

### 2.8 Statistical analysis

Statistical analysis were performed with SPSS software (Version 24; SPSS Inc., Chicago, Ill., USA). For the *in vitro* experiment, a two-way analysis of variance (ANOVA) was conducted to analyze the effects of PEG-6000 treatment and fungal species on the growth and physiological responses of the four fungi. For the pot experiment, two-way ANOVA examined the effects of drought treatment and fungal inoculation on the growth and physiological responses of *P. tabuliformis* seedlings. Mean values were compared using Duncan’s multiple range test at a significance level of *p* < 0.05. Correlation analyses were conducted to assess the influence of different fungal inoculants on plant parameters. Principal component analysis (PCA) was employed to analyze characteristics including fungal colonization rates, plant biomass, enzyme activities, and nutrient element contents. All graphs were generated using GraphPad Prism software (version 10.1.0).

## 3 Results

### 3.1 Effects of different concentrations of PEG-6000 on the biomass of root symbiotic fungi

**Figure 1:**
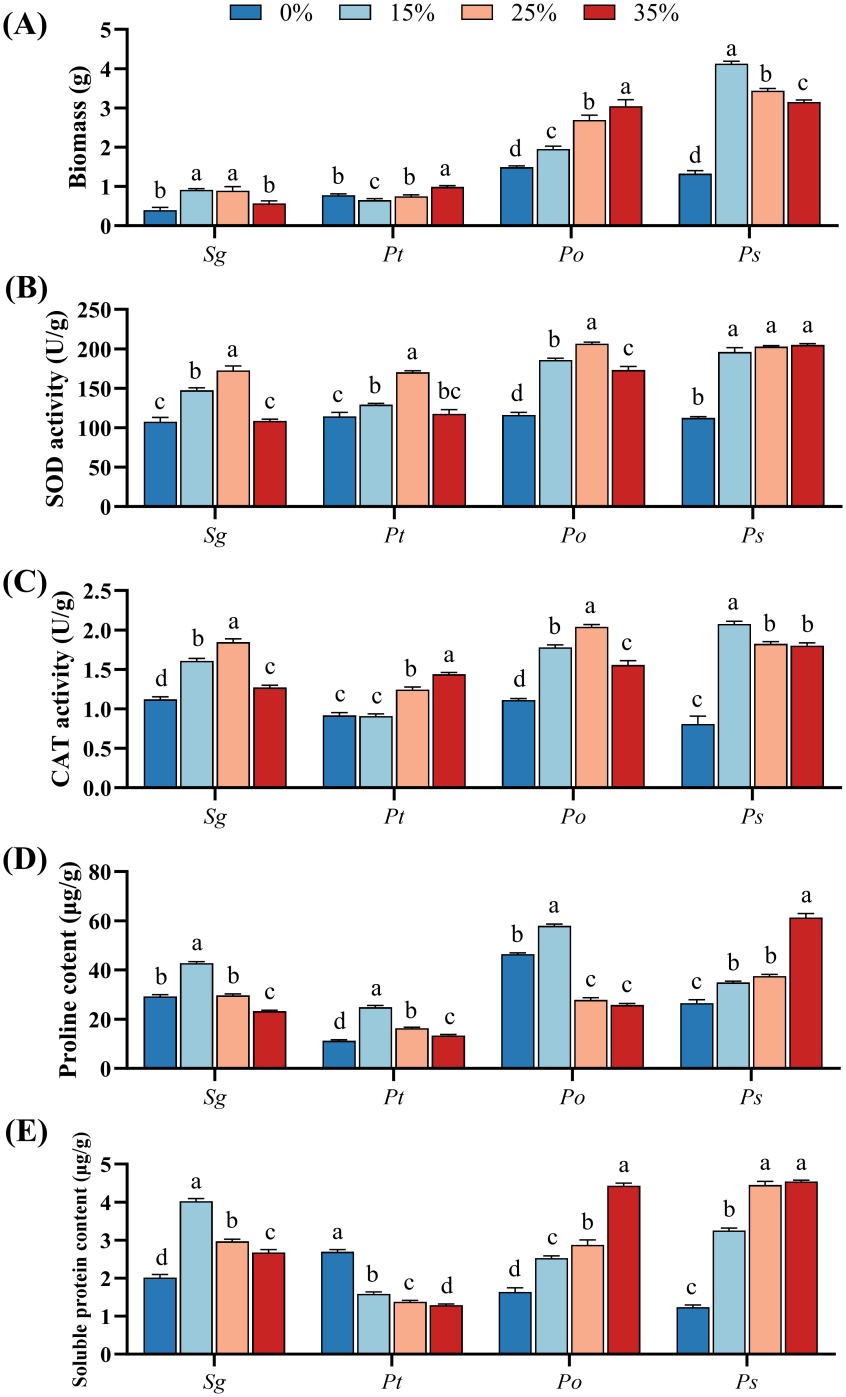
Effects of different concentrations of PEG-6000 (0%, 15%, 25%, and 35%) on the biomass **(A)**, superoxide dismutase activity **(B)**, catalase activity **(C)**, proline **(D)** and soluble protein **(E)** of *Suillus granulatus* (*Sg*), *Pisolithus tinctorius* (*Pt*), *Pleotrichocladium opacum* (*Po*), and *Pseudopyrenochaeta* sp. (*Ps*). Data (means ± SD, n = 3) are significantly different (*p* < 0.05) if followed by different letters above the bars.

The two-way ANOVA results (**Supplementary Table S1**) revealed significant effects of PEG stress, fungal species, and their interaction on the biomass of the four root symbiotic fungi. The biomass of the *Sg* and *Ps* strains initially increased and then decreased with increasing PEG-6000 concentration, reaching a maximum under the 15% PEG-6000 treatment in *Ps* strain, and 15% and 25% PEG-6000 treatments in *Sg* strain (**Figure 1A**). In contrast, the biomass of the *Pt* strain first decreased and then increased, with a 27.0% increase under the 35% PEG-6000 treatment compared to the 0% treatment. The biomass of the *Po* strain showed a gradual increase with rising PEG-6000 concentrations and reached the highest under 35% PEG-6000 treatment. Overall, the biomass accumulation of the *Sg* strain was higher than that of the *Pt* strain, and the *Ps* strain had greater biomass accumulation than the *Po* strain across all PEG-6000 concentrations.

### 3.2 Effects of different concentrations of PEG-6000 on the antioxidant enzyme activities of root symbiotic fungi

With increasing PEG-6000 concentrations, both SOD and CAT activities in the *Sg* and *Po* strains increased, reaching their maximum under the 25% PEG-6000 treatment (**Figures 1B, C**). Compared to the control treatment, the SOD activity in the *Pt* strain significantly increased under the 15% and 25% PEG-6000 treatments, reaching its peak at 25% PEG-6000. The CAT activity in the *Pt* strain showed no significant difference from the control at 15% PEG-6000 but significantly increased under the 25% and 35% PEG-6000 treatments, with the highest activity observed at 35% PEG-6000. In contrast, the SOD and CAT activities in the *Ps* strain remained significantly higher under all PEG-6000 treatments compared to the 0% PEG-6000 treatment. Overall, the two dark septate endophytes (*Po* and *Ps*) exhibited greater improvements in antioxidant enzyme activities in response to PEG-6000 stress than the two ectomycorrhizal fungi (*Sg* and *Pt*).

### 3.3 Effects of different PEG-6000 concentrations on osmotic adjustment substances in symbiotic root fungi

All four fungi influenced the accumulation of proline and soluble proteins in response to drought stress induced by PEG-6000 (**Figures 1D, E**). For the *Sg*, *Pt*, and *Po* strains, proline content peaked at the 15% PEG-6000 treatment, significantly higher than the control, and subsequently decreased with increasing PEG-6000 concentrations. In contrast, PEG-6000 stress consistently and significantly increased proline levels in the *Ps* strain, with no significant difference between the 15% and 25% treatments. However, at 35% PEG-6000, the *Ps* strain exhibited significantly higher proline content compared to the other treatments (**Figure 1D**).

PEG-6000 stress consistently and significantly increased soluble protein levels in the *Sg*, *Po*, and *Ps* strains, while it resulted in a significant reduction in the *Pt* strain. The *Sg* strain reached its maximum soluble protein content at 15% PEG-6000, followed by a decreasing trend with increasing PEG-6000 concentrations. Conversely, soluble protein content in the *Po* and *Ps* strains increased significantly with rising PEG-6000 levels, peaking at 35% PEG-6000. In contrast, the *Pt* strain exhibited the lowest soluble protein content at 35% PEG-6000 (**Figure 1E**).

### 3.4 Root fungal colonization and growth performance of *P. tabuliformis*

**Figure 2:**
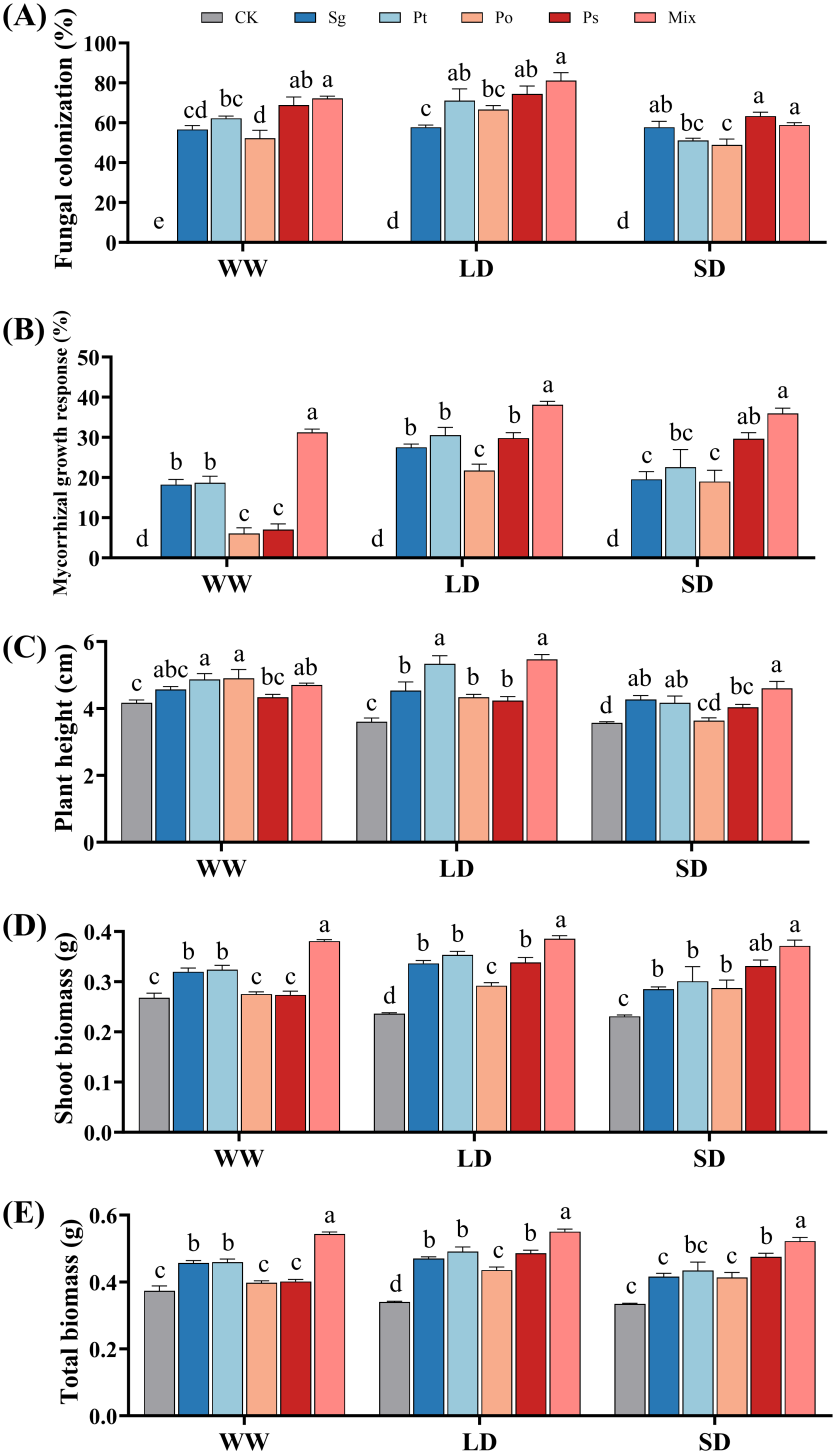
Effects of different inoculation treatments on the growth performance of *Pinus tabuliformis* seedlings under drought stress. Data (means ± SD, n = 3) are significantly different (*p* < 0.05) if followed by different letters above the bars. **(A)**, fungal colonization rate; **(B)**, mycorrhizal growth response; **(C)**, plant height; **(D)**, shoot biomass and **(E)**, total biomass. *Sg*, *Suillus granulatus*; *Pt*, *Pisolithus tinctorius*; *Po*, *Pleotrichocladium opacum*; *Ps*, *Pseudopyrenochaeta* sp.; Mix, mixed inoculation of four root symbiotic fungi. WW, well-watered; LD, light drought; SD, severe drought.

In the non-inoculated treatments, no fungal structure was observed in the roots of *P. tabuliformis* (**Figure 2A**). The fungal colonization rates showed that, except for the *Po* inoculation under SD treatment, all other inoculation treatments under various drought conditions had colonization rates exceeding 50% (**Figure 2A**). The colonization rates of *Sg* increased with drought severity, whereas the rates for other treatments first increased and then decreased. The colonization rates for *Pt*, *Po*, and mix treatments peaked under LD conditions. The mix treatment had higher colonization rates than other treatments under WW and LD conditions.

There was a significant interaction between fungal inoculation and drought stress on the mycorrhizal growth response of the seedlings (**Supplementary Table S2**). With increasing drought stress, the mycorrhizal growth response values for the inoculation treatments initially increased and then decreased (**Figure 2B**). All inoculation treatments positively affected the growth of *P. tabuliformis* seedlings under drought conditions. The MGR values of the mixed inoculation treatment were higher than those of individual inoculations, indicating the greatest growth promotion effects under drought stress.

The plant height of non-inoculated, *Sg*-, *Po*-, and *Ps*-inoculated seedlings decreased with increasing drought stress (**Figure 2C**). The plant height of *Pt*- and mix-inoculated seedlings first increased and then decreased, peaking under LD conditions. Shoot and total biomass of non-inoculated seedlings decreased with increasing drought stress, while inoculated seedlings showed an initial increase followed by a decrease, reaching a maximum under LD conditions (**Figures 2D, E**). Under all drought conditions, mixed inoculation treatments resulted in significantly higher shoot and total biomass compared to single inoculations.

### 3.5 Effects of different inoculation treatments on the antioxidant enzyme activities of *P. tabuliformis* seedlings under drought stress

**Figure 3:**
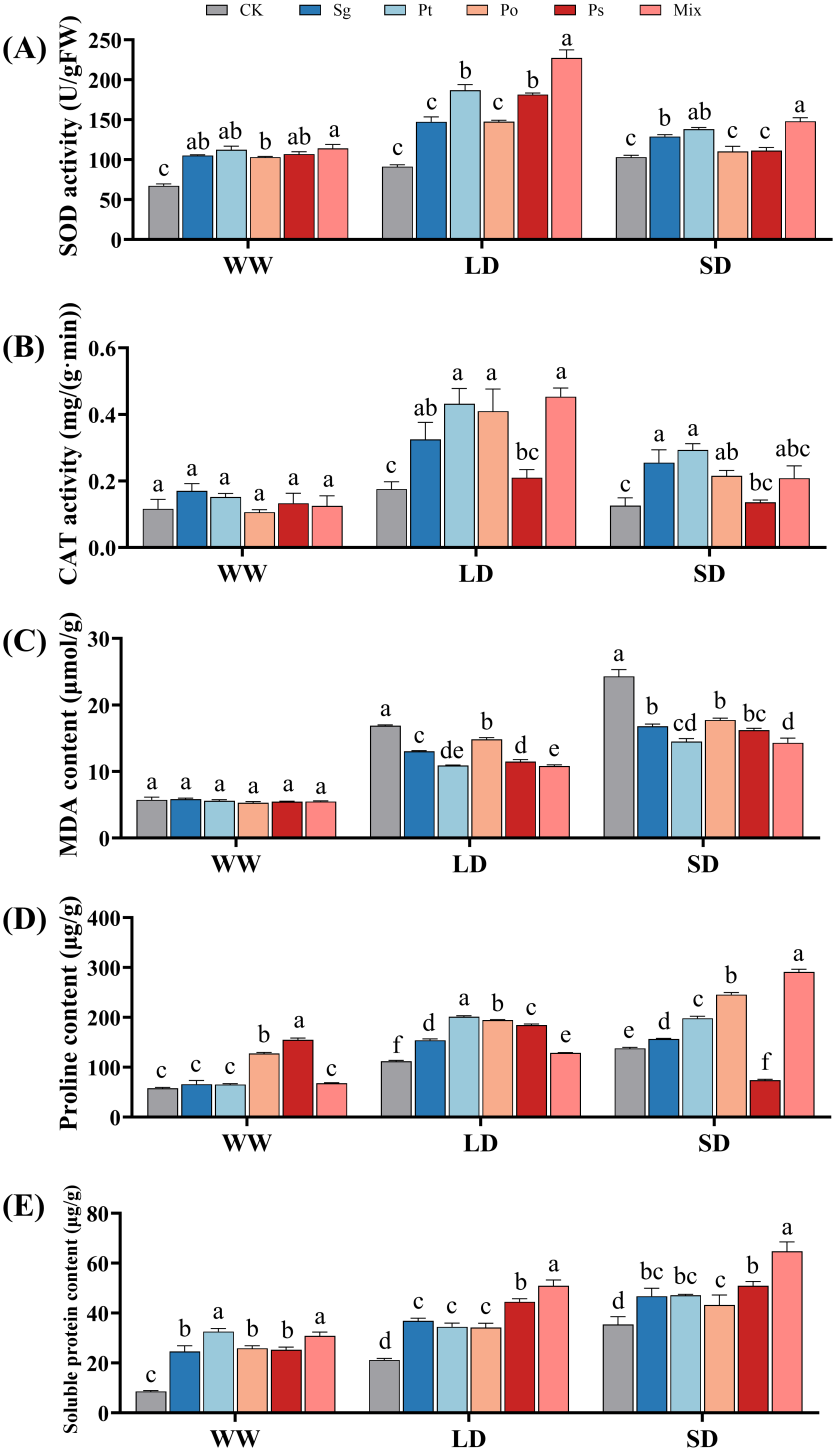
Effects of different inoculation treatments on the physiological characteristics of *Pinus tabuliformis* seedlings under drought stress. Data (means ± SD, n = 3) are significantly different (*p* < 0.05) if followed by different letters above the bars. **(A)**, superoxide dismutase activity; **(B)**, catalase activity; **(C)**, malondialdehyde content; **(D)**, proline and **(E)**, soluble protein. *Sg*, *Suillus granulatus*; *Pt*, *Pisolithus tinctorius*; *Po*, *Pleotrichocladium opacum*; *Ps*, *Pseudopyrenochaeta* sp.; Mix, mixed inoculation of four root symbiotic fungi. WW, well-watered; LD, light drought; SD, severe drought.

With increasing drought stress, the SOD activity of non-inoculated seedlings gradually increased, while that of inoculated seedlings first increased and then decreased (**Figure 3A**). The SOD activity of all inoculated seedlings peaked under LD conditions. CAT activity in seedlings from all treatments increased first and then decreased with increasing drought stress severity (**Figure 3B**). Under LD and SD treatments, inoculated seedlings had higher SOD and CAT activities than non-inoculated ones, indicating that inoculation treatments enhanced antioxidant enzyme activities to cope with drought stress.

### 3.6 Effects of different inoculation treatments on the MDA content of *P. tabuliformis* seedlings under drought stress

Under drought stress, the MDA content of seedlings increased with drought severity, while inoculation treatments reduced MDA accumulation (**Figure 3C**). There was no significant difference in MDA content between inoculated and control seedlings under WW conditions. Under LD conditions, inoculation treatments significantly reduced MDA content by 22.8%, 35.4%, 12.0%, 32.0%, and 35.9%, respectively. Under SD conditions, the reductions were 30.9%, 40.5%, 27.0%, 33.3%, and 41.2%, respectively. mixed inoculation treatments significantly reduced MDA content under LD and SD conditions.

### 3.7 Effects of different inoculation treatments on the osmotic adjustment substance contents of *P. tabuliformis* seedlings under drought stress

With increasing drought severity, the proline content of non-inoculated, *Sg*-, *Po*-, and mix-inoculated seedlings gradually increased, while the proline content of *Pt*- and *Ps*-inoculated seedlings first increased and then decreased (**Figure 3D**). Under SD conditions, the proline content of all inoculation treatments significantly increased compared to the control, except for the *Ps* inoculation. The mix-inoculated treatment had the highest proline content.

Under WW, LD, and SD conditions, all fungal inoculation treatments significantly increased the soluble protein content in the seedlings (**Figure 3E**). Specifically, under WW conditions, the soluble protein content in the mix and *Pt* treatments was significantly higher than in the *Sg*, *Po*, and *Ps* treatments. Under LD and SD conditions, the soluble protein content of the mixed-inoculated seedings was significantly higher than that of the other four single inoculation treatments.

### 3.8 Effects of different inoculation treatments on the nutrient content of *P. tabuliformis* seedlings under drought stress

**Figure 4:**
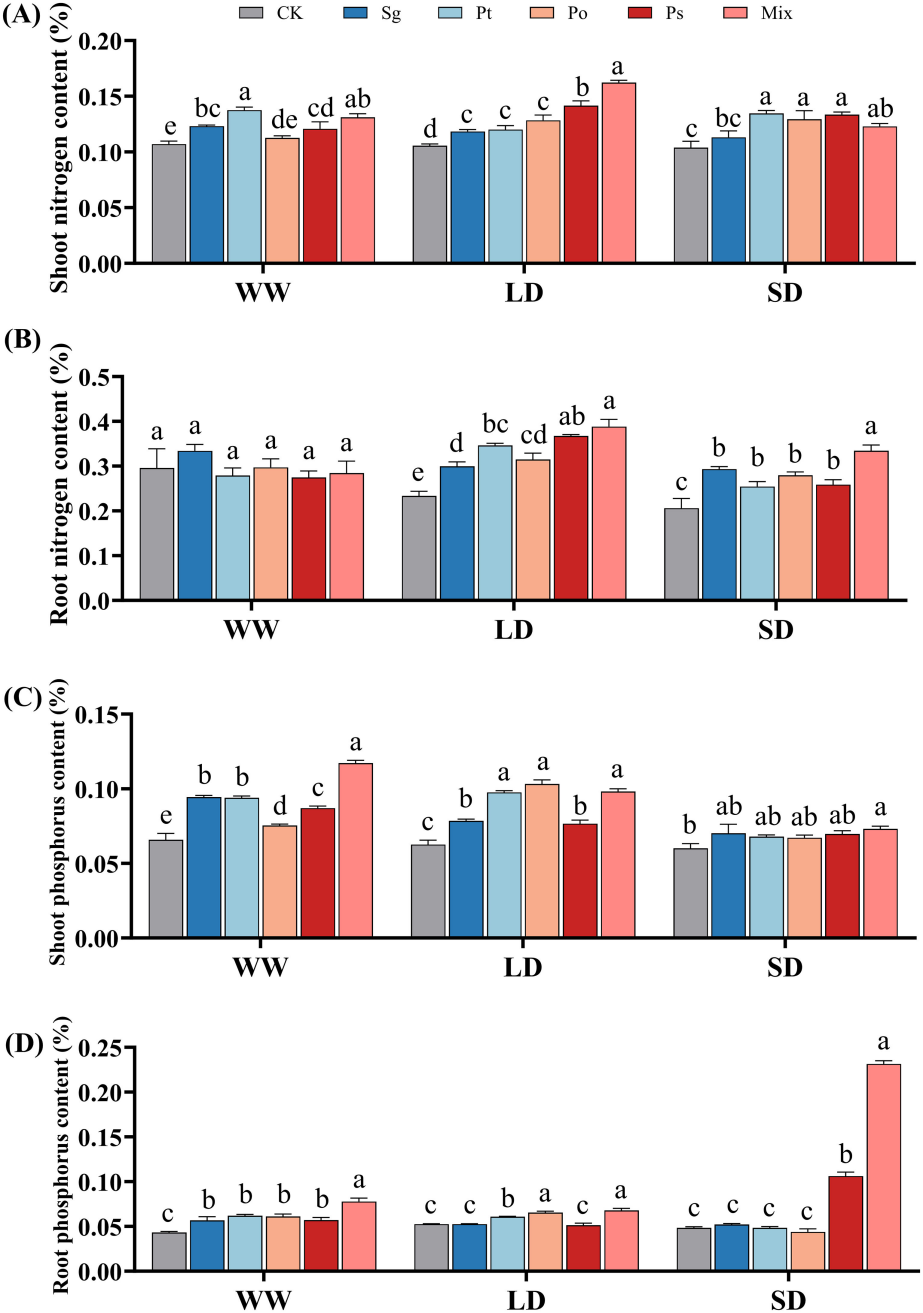
Effects of different inoculation treatments on nitrogen and phosphorus content in the shoots and roots of *Pinus tabuliformis* seedlings under drought stress Data (means ± SD, n = 3) are significantly different (*p* < 0.05) if followed by different letters above the bars. **(A)**, shoot nitrogen content; **(B)**, root nitrogen content; **(C)**, shoot nitrogen content and **(D)**, root phosphorus content. *Sg*, *Suillus granulatus*; *Pt*, *Pisolithus tinctorius*; *Po*, *Pleotrichocladium opacum*; *Ps*, *Pseudopyrenochaeta* sp.; Mix, mixed inoculation of four root symbiotic fungi. WW, well-watered; LD, light drought; SD, severe drought.

With increasing drought severity, the N content in the shoots and roots of non-inoculated and *Sg*-inoculated seedlings gradually decreased, while N content in the shoots and roots of *Ps*- and mix-inoculated seedlings first increased and then decreased (**Figures 4A, B**). The P content in the shoots of non-inoculated seedlings gradually decreased, while the P content in the roots first increased and then decreased (**Figures 4C, D**). The P content in the shoots and roots of *Sg*-, *Ps*-, and mix-inoculated seedlings gradually decreased, whereas in *Po*-inoculated seedlings, it first increased and then decreased with increasing drought severity (**Figures 4C, D**).

### 3.9 Correlation analysis

**Figure 5:**
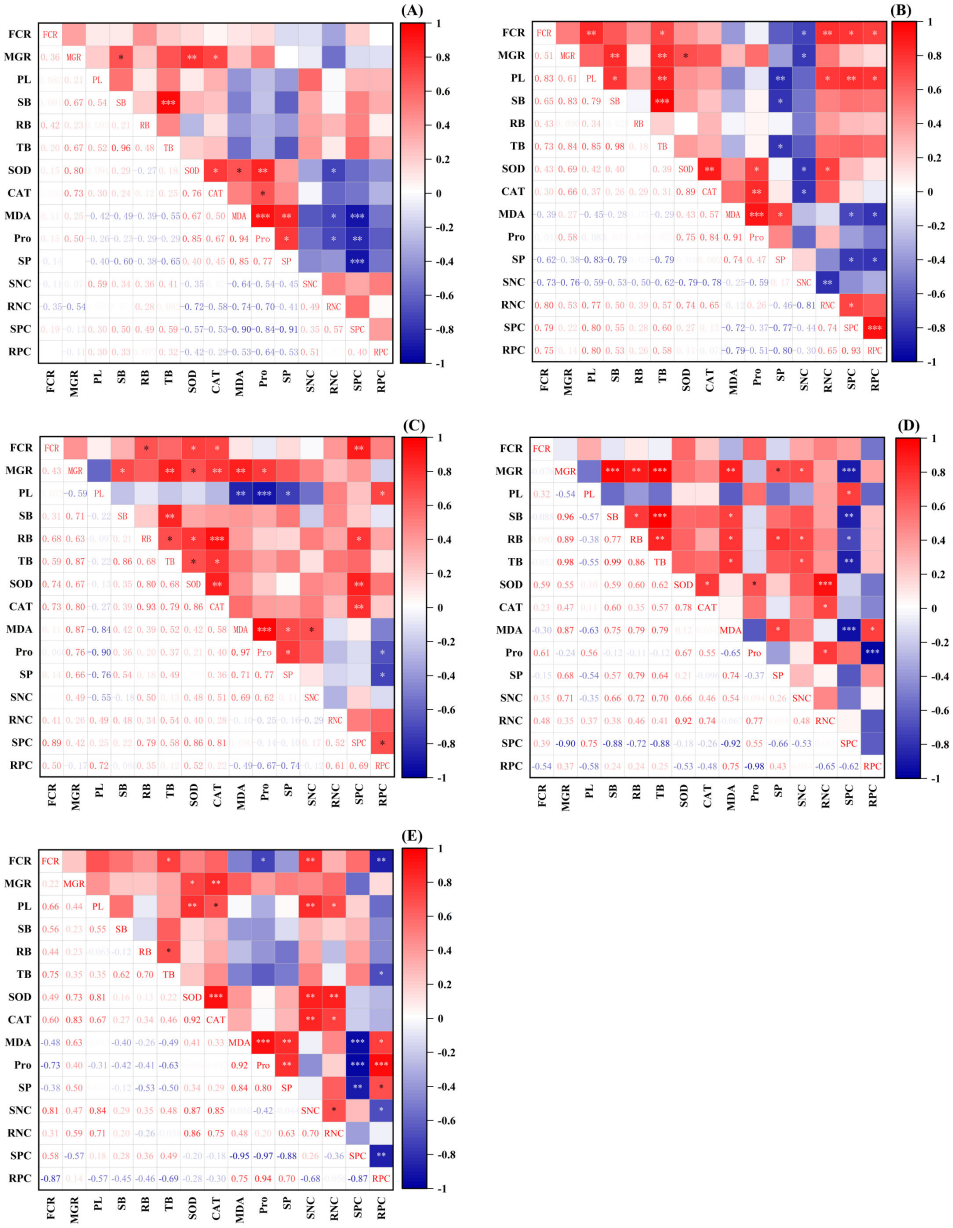
Correlations among indicators in the pot experiment. **(A)** *Sg*, **(B)** *Pt*, **(C)** *Po*, **(D)** *Ps*, **(E)** Mix; *: (*p* < 0.05); **: *p* < 0.01; ***: *p* < 0.001; FCR, Fungal colonization rate; MGR, Mycorrhizal growth response; PL, Plant height; SB, Shoot biomass; RB, Root biomass; TB, Total biomass; SOD, Superoxide dismutase activity; CAT, Catalase activity; MDA, Malondialdehyde content; Pro, Proline; SP, Soluble protein; SNC, Shoot N content; RNC, Root N content; SPC, Shoot P content; RPC, Root P content.

Correlation analysis of different inoculation treatments on the growth and physiological indexes of *P. tabuliformis* seedlings under drought stress revealed significant positive correlations between fungal colonization rates and various growth and nutrient parameters (**Figure 5**). The colonization rates of *Pt*-inoculated seedlings were significantly positively correlated with plant height, total biomass, root N content, shoot P content, and root P content (*p* < 0.05) (**Figure 5B**). *Po*-inoculated seedlings showed significant positive correlations with root biomass, SOD, CAT, and shoot P content (*p* < 0.05) (**Figure 5C**). Mix-inoculated seedlings exhibited significant positive correlations with total biomass and shoot N content (*p* < 0.05) (**Figure 5E**). Higher fungal colonization rates of *Pt*-, *Po*-, and mix-inoculated seedlings promoted seedling growth and nutrient absorption.

The root biomass of *Po*-inoculated seedlings was significantly positively correlated with SOD, CAT, and shoot P content, while total biomass was significantly positively correlated with SOD and CAT (*p* < 0.05) (**Figure 5C**). *Ps*-inoculated seedlings’ root biomass was significantly positively correlated with soluble protein and shoot N content (*p* < 0.05) (**Figure 5D**). The plant height of mix-inoculated seedlings was significantly positively correlated with SOD, CAT, shoot N content, and root N content (*p* < 0.05) (**Figure 5E**). These results indicate that the strong antioxidant defense and nutrient uptake abilities of *Po*- and mix-inoculated seedlings significantly promote growth and improve drought resistance.

### 3.10 PCA analysis

**Figure 6:**
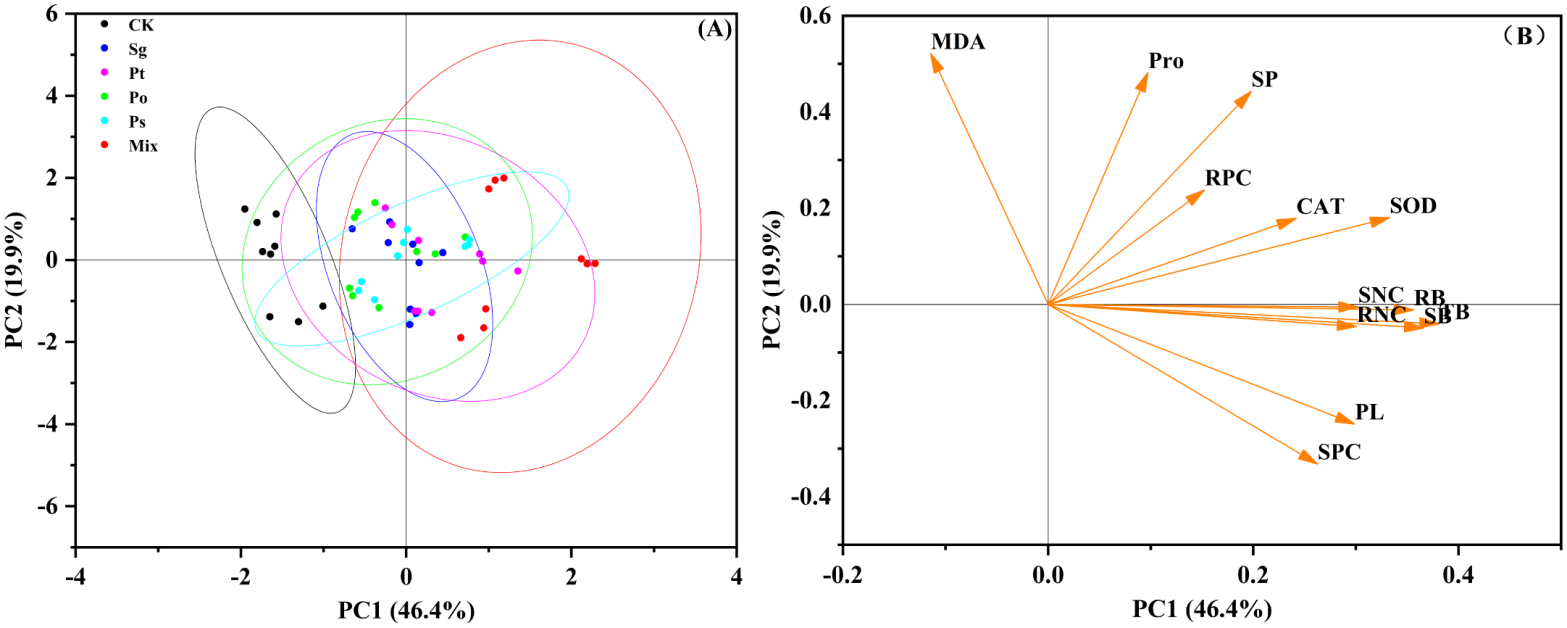
Principal component analysis (PCA) of indicators in the pot experiment. **(A)** *Sg* indicates *Suillus granulatus*; *Pt* indicates *Pisolithus tinctorius*; *Po* indicates *Pleotrichocladium opacum*; *Ps* indicates *Pseudopyrenochaeta* sp.; Mix indicates mixed inoculation of four root symbiotic fungi.; **(B)** PL, Plant height; SB, Shoot biomass; RB, Root biomass; TB, Total biomass; SOD, Superoxide dismutase activity; CAT, Catalase activity; MDA, Malondialdehyde content; Pro, Proline; SP, Soluble protein; SNC, Shoot N content; RNC, Root N content; SPC, Shoot P content; RPC, Root P content.

PCA was used to evaluate the similarity among different inoculation treatments and the relationships between plant height, biomass, enzyme activity, antioxidant capacity, and nutrient composition (**Figure 6**). The first principal component (PC1) and the second principal component (PC2) explained 46.4% and 19.9% of the variance, respectively. There were significant differences between control and inoculated treatments under drought stress (**Figure 6A**). The mix inoculation treatment was significantly separated from other treatments. Antioxidant enzyme activity, osmotic adjustment substances, and nutrient content were the main factors affecting plant growth and were positively correlated with biomass (**Figure 6B**). The results indicated that inoculation treatments promoted plant growth and improved antioxidant capacity, enhancing the tolerance of *P. tabuliformis* seedlings to drought stress.

## 4 Discussion

### 4.1 Effects of drought stress on the growth and physiological characteristics of root symbiotic fungi

The biomass of fungi is a crucial indicator of their resistance under stressful environments. All four fungi exhibited normal growth under PEG stress, indicating their tolerance to PEG-induced drought stress. Abiotic stresses, such as drought and salt, have been shown to induce an increase in plant peroxisome content (Fahy et al., 2017; Castillo et al., 2008; Sanad et al., 2019). Li et al. (2022b) demonstrated that strains with greater drought resistance exhibit significant increases in antioxidant enzyme activities (SOD, POD, and CAT) under drought stress, indicating that increased antioxidant enzyme activity is a response to drought in sensitive strains. In this study, the biomass of the *Sg*, *Po*, and *Ps* strains, except for the *Pt* strain, was greater under other PEG-6000 treatments compared to the 0% PEG-6000 treatment. This may be related to the higher contents of antioxidant enzymes and osmotic adjustment substances in these strains under PEG-6000 treatments. SOD and CAT are critical protective enzymes in the antioxidant system, responsible for scavenging reactive oxygen species (ROS) and H_2_O_2_, working together to maintain the balance between ROS production and scavenging (Raja et al., 2017). This study found that the activities of antioxidant enzymes and the contents of osmotic adjustment substances increased in all four fungi under PEG stress, with SOD and CAT activities showing consistent changes. This indicates that all four fungi could alleviate oxidative damage by synthesizing antioxidant enzymes to scavenge ROS produced by PEG-6000 stress. Simultaneously, they improved their accumulation of proline and soluble proteins, promoting cellular osmoregulation to protect mycelial cell membrane structures, thereby enhancing stress tolerance. This study also revealed that DSE strains had greater biomass than ECMF strains, suggesting that DSE strains were less affected by PEG-6000 stress, possibly due to their highly melanized hyphae. Melanin in fungi serves to protect them from harmful environmental conditions (Fernandez and Koide, 2013).

### 4.2 Effects of root symbiotic fungi on the growth and development of *P. tabuliformis* seedlings under drought stress

Microorganisms are natural partners in plant defense mechanisms under adverse conditions (Meena et al., 2017). In this study, typical mycorrhizal structures and DSE hyphae and microsclerotia were observed in root samples inoculated with ECMF and DSE strains, indicating effective colonization even under drought conditions. Additionally, the mycorrhizal growth response values of inoculation treatments were all positive under drought stress, with mixed inoculation treatments showing greater effects than individual treatments. Among the four individual inoculation treatments, the Ps-inoculated seedlings under SD conditions exhibited higher fungal colonization rates, mycorrhizal growth responses, and biomass compared to those inoculated with the other three fungi. This increased performance is likely due to the reduction in MDA content and the increase in soluble protein content in the Ps-inoculated seedlings, which enhances cell membrane stability and improves dehydration resistance, allowing the plants to better adapt to stressful environments. These findings align with previous pure culture experiments where the Ps strain demonstrated higher biomass and stronger drought tolerance under the highest PEG concentration stress. Thus, different fungi exhibit varying levels of drought tolerance and influence the drought resistance of plants differently. Studies have shown that inoculation with *Amanita vaginata* significantly promotes the growth of *P. tabuliformis* (Zhang et al., 2011), and the biomass of *Hedysarum scoparium* inoculated with DSE under drought stress was significantly greater than that of non-inoculated treatments (Li et al., 2019). However, few studies have investigated the co-inoculation of ECMF and DSE strains under drought stress. This experiment demonstrated that inoculating ECMF and DSE strains under drought stress promoted increases in plant height, shoot biomass, root biomass, and total biomass of *P. tabuliformis* seedlings. These growth indices were significantly higher in mixed inoculation treatments compared to non-inoculated and individual inoculation treatments. Increased plant biomass indicates more organic matter to support plants, enhancing their survival in arid environments. Our study revealed that inoculating root symbiotic fungi under drought stress effectively mitigated adverse environmental effects on *P. tabuliformis* seedlings, promoting growth and improving drought resistance. Furthermore, mixed inoculation treatments had synergistic rather than competitive effects on the growth of *P. tabuliformis* seedlings under drought conditions.

### 4.3 Effects of root symbiotic fungi on the physiological characteristics of *P. tabuliformis* seedlings under drought stress

Drought stress leads to the overproduction of ROS in plants, causing degradation of lipids, proteins, and nucleic acids, damaging plant cells, and reducing growth and development (Gill and Tuteja, 2010; Kapoor et al., 2019). Plants produce enzymes such as SOD and CAT to scavenge ROS, protecting cells from oxidative damage and preserving membrane integrity (Wu et al., 2006; Raja et al., 2017). In this study, antioxidant enzyme activities in *P. tabuliformis* seedlings inoculated with root symbiotic fungi increased under drought stress, indicating that these fungi protect host plants from oxidative damage by enhancing antioxidant enzyme activities. Under LD conditions, mixed inoculation treatments significantly increased SOD and CAT activities in seedlings compared to individual treatments, indicating a greater ability to eliminate ROS. MDA content, an indicator of oxidative damage, reflects the degree of stress injury to plants. Excessive MDA accumulation often results from oxidative damage to membrane lipids, with lower MDA levels indicating higher membrane stability (Quiroga et al., 2020). In this study, MDA content in *P. tabuliformis* seedlings decreased after inoculation with root symbiotic fungi, indicating improved cell membrane stability. Under LD and SD conditions, mixed inoculation treatments resulted in significantly lower MDA content compared to control and individual inoculation treatments, consistent with findings in *P. sylvestris* var. *mongolica* seedlings (Zhao et al., 2020). However, under WW conditions, there was no significant difference in MDA content between mixed inoculation and control treatments, indicating no significant synergistic effect of co-inoculation under well-watered conditions.

Osmotic adjustment is a crucial mechanism for plants to regulate water potential under drought stress. Proline and soluble protein are key osmotic adjustment substances that allow plants to adapt to stressful environments by increasing dehydration tolerance through accumulation (Pal et al., 2018; Sharma and Verslues, 2010; Azmat and Moin, 2019). In this study, proline content in inoculated seedlings under LD and SD conditions was significantly higher than in control treatments, with mixed inoculation treatments showing the highest proline content under SD conditions. Rezaei-Chiyaneh et al. (2021) also found that co-inoculated plants under severe water stress had the highest proline content. This study indicates that co-inoculation of ECMF and DSE strains promotes proline biosynthesis, enhancing osmotic regulation under severe water stress. Higher contents of soluble protein in inoculated plants suggest greater osmotic ability, helping seedlings maintain turgor pressure under drought conditions (Liu et al., 2021). Therefore, root symbiotic fungi improve osmotic balance by increasing the content of osmotic adjustment substances, enhancing plant tolerance and alleviating the negative effects of drought.

Mycorrhizal symbionts significantly affect the uptake of nitrogen and phosphorus by plants (Veresoglou et al., 2012). Research has shown that fungal inoculation improves nutrient recovery efficiency, accumulation, and growth in host plants (Surono and Narisawa, 2017; Vergara et al., 2017, 2018; Lu et al., 2016). In this experiment, N contents in the shoots and roots of inoculated plants under LD and SD conditions were significantly higher than in control treatments, with mixed inoculation treatments showing the highest N contents under LD conditions. Mycorrhizal fungi increase nitrogen uptake under drought stress by enhancing root hydraulic conductivity (Graham and Syversen, 1984). Mycorrhizal fungi also promote nitrogen uptake by decomposing organic matter (Hodge et al., 2001; Goussous and Mohammad, 2009), helping maintain water status under water scarcity. Mixed inoculation treatments significantly increased P content in shoots and roots under WW conditions and were more effective than individual treatments under LD and SD conditions. Fungi can explore more soil volume than non-mycorrhizal plants, enhancing P uptake (Evelin et al., 2009). Adding mycorrhizal fungi to soil increases microbial biomass and CO_2_ release, forming weak acids that dissolve phosphorus-bearing minerals, and increasing phosphorus availability (Subramanian et al., 2006). Thus, plants inoculated with mycorrhizal fungi exhibit higher productivity even under adverse conditions. In summary, root symbiotic fungi improve the uptake of nutrients such as N and P, promoting growth and enhancing plant resistance. The synergistic effects of ECMF and DSE on nutrient absorption are greater than individual inoculations.

## 5 Conclusion

Drought stress affects a range of physiological changes in plants, and root symbiotic fungi can help plants withstand the adverse effects of drought. This study demonstrated that root symbiotic fungi (*Sg*, *Po*, *Ps*, and *Pt*) enhance the growth and drought resistance of *P. tabuliformis* seedlings. All fungi tolerated PEG-induced drought stress, with increased antioxidant enzyme activities and osmotic adjustment substances. DSE strains showed greater resilience due to their melanized hyphae. Acids produced by root symbiotic fungi under drought stress may help plants to activate elements in the soil that plants fail to take up. Therefore, the determination of the composition and content of metabolites of root symbiotic fungi under drought stress could be carried out in future studies. Inoculation, especially mixed inoculation, significantly improved plant height, biomass, and drought resistance. The fungi increased antioxidant enzymes and osmotic substances, mitigating oxidative damage and enhancing osmotic regulation. Additionally, they improved nutrient uptake, particularly nitrogen and phosphorus. These findings suggest that root symbiotic fungi, particularly in combination, are effective in enhancing drought resistance and growth of *P. tabuliformis*, providing valuable insights for afforestation and forest management in arid regions.

## Data Availability

The original contributions presented in the study are included in the article/**Supplementary Material**. Further inquiries can be directed to the corresponding author.

## References

[B1] Addy H. D. Piercey M. M. Currah R. S. (2005). Microfungal endophytes in root. Can. J. Bot. 83, 1–13. doi: 10.1139/b04-171

[B2] Alori E. T. Emmanuel O. C. Glick B. R. Babalola O. O. (2020). Plant-archaea relationships: a potential means to improve crop production in arid and semi-arid regions. World J. Microb. Biot. 36, 133. doi: 10.1007/s11274-020-02910-6 32772189

[B3] Azmat R. Moin S. (2019). The remediation of drought stress under VAM inoculation through proline chemical transformation action. J. Photochem. Photobiol. 193, 155–161. doi: 10.1016/j.jphotobiol.2019.03.002 30884285

[B4] Bao S. D. (2000). Agrochemical Analysis of Soil (Beijing: Chinese Agricultural Press), 44–49.

[B5] Bates L. S. Waldren R. P. Teare I. D. (1973). Rapid determination of proline for water stress studies. Plant Soil 39, 205–207. doi: 10.1007/BF00018060

[B6] Brundrett M. Bougher N. Dell B. Grove T. Malajczuk N. (1996). Working with Mycorrhizas in Forestry and Agriculture. (Canberra: Australian Centre for International Agricultural Research). doi: 10.13140/2.1.4880.5444

[B7] Campion E. M. Loughran S. T. Walls D. (2017). Protein quantitation andanalysis of purity. Methods Mol. Biol. 1485, 225–255. doi: 10.1007/978-1-4939-6412-3_12 27730556

[B8] Castillo M. C. Sandalio L. M. Del Rio L. A. Leon J. (2008). Peroxisome proliferation, wound-activated responses and expression of peroxisome-associated genes are cross-regulated but uncoupled in Arabidopsis thaliana. Plant Cell Environ. 31, 492–505. doi: 10.1111/j.1365-3040.2008.01780.x 18194426

[B9] Chen Z. J. Zhang X. L. He X. Y. Davi N. K. Li L. L. Bai X. P. (2015). Response of radial growth to warming and CO_2_ enrichment in southern Northeast China:a case of. Clim. Change 130, 559–571. doi: 10.1007/s10584-015-1356-8

[B10] Chidumayo E. N. (2008). Implications of climate warming on seedling emergence and mortality of African savanna woody plants. Plant Ecol. 198, 61–71. doi: 10.1007/s11258-007-9385-7

[B11] Chu H. Wang C. Li Z. Wang H. Xiao Y. Chen J.. (2019). The dark septate endophytes and ectomycorrhizal fungi effect on *Pinus tabulaeformis* Carr. seedling growth and their potential effects to pine wilt disease resistance. Forests 10, 140. doi: 10.3390/f10020140

[B12] Chu H. Wang C. Wang H. Chen H. Tang M. (2016). Pine wilt disease alters soil properties and root-associated fungal communities in *Pinus tabulaeformis* forest. Plant Soil 404, 237–249. doi: 10.1007/s11104-016-2845-x

[B13] Chu H. Wang H. Zhang Y. Li Z. Wang C. Dai D.. (2021). Inoculation with ectomycorrhizal fungi and dark septate endophytes contributes to the resistance of Pinus spp. to pine wilt disease. Front. Microbiol. 12, 687304. doi: 10.3389/FMICB.2021.687304 34421845 PMC8377431

[B14] Cook B. I. Smerdon J. E. Seager R. Coats S. (2014). Global warming and 21st century drying. Clim. Dyn. 43, 2607–2627. doi: 10.1007/s00382-014-2075-y

[B15] Corrales A. Koch R. A. Vasco-Palacios A. M. Smith M. E. Ge Z. W. Henkel T. W. (2022). Diversity and distribution of tropical ectomycorrhizal fungi. Mycologia 114, 11–15. doi: 10.1080/00275514.2022.2115284 36194092

[B16] Deng X. Song X. S. Yin D. C. Song R. Q. (2017). Effect of inoculating *Phialocephala fortinii* D575 and *Suillus luteus* N94 on the growth of *Pinus sylvestris* var. *mongolica* and its resistant to damping-off. For. Pest Dis. 36, 21–25. doi: 10.3969/j.issn.1671-0886.2017.01.006 (in Chinese)

[B17] Elavarthi S. Martin B. (2010). Spectrophotometric assays for antioxidant enzymes in plants. Methods Mol. Biol. 639, 273–280. doi: 10.1007/978-1-60761-702-0_16 20387052

[B18] Evelin H. Kapoor R. Giri B. (2009). Arbuscular mycorrihizal fungi in alleviation of salt stress: a review. Ann. Bot. 104, 1263–1280. doi: 10.1093/aob/mcp251 19815570 PMC2778396

[B19] Fahy D. Sanad M. N. Duscha K. Lyons M. Liu F. Bozhkov P.. (2017). Impact of salt stress, cell death, and autophagy on peroxisomes: quantitative and morphological analyses using small fluorescent probe N-BODIPY. Sci. Rep. 7, 1–18. doi: 10.1038/srep39069 28145408 PMC5286434

[B20] Farrell C. Szota C. Arndt S. K. (2017). Does the turgor loss point characterize drought response in dryland plants? Plant Cell Environ. 40, 1500–1511. doi: 10.1111/pce.12948 28342210

[B21] Fernandez C. W. Koide R. T. (2013). The function of melanin in the ectomycorrhizal fungus Cenococcum geophilum under water stress. Fungal Ecol. 6, 479–486. doi: 10.1016/j.funeco.2013.08.004

[B22] Gaber D. A. Berthelot C. Blaudez D. Kovács G. M. Franken P. (2023). Impact of dark septate endophytes on salt stress alleviation of tomato plants. Front. Microbiol. 14, 1124879. doi: 10.3389/fmicb.2023.1124879 37415811 PMC10320394

[B23] Gao J. Wang J. F. Li Y. H. (2023). Effects of soil nutrients on plant nutrient traits in natural *Pinus tabuliformis* forests. Plants 12, 735–735. doi: 10.3390/PLANTS12040735 36840084 PMC9967982

[B24] Gill S. S. Tuteja N. (2010). Reactive oxygen species and antioxidant machinery in abiotic stress tolerance in crop plants. Plant Physiol. Bioch. 48, 909–930. doi: 10.1016/j.plaphy.2010.08.016 20870416

[B25] Gleason F. H. Midgley D. J. Letcher P. M. McGee P. A. (2006). Can soil Chytridiomycota survive and grow in different osmotic potentials. Mycol. Res. 110, 869–875. doi: 10.1016/j.mycres.2006.04.002 16876703

[B26] Goussous S. J. Mohammad M. J. (2009). Comparative effect of two arbuscular mycorrhizae and N and P fertilizers on growth and nutrient uptake of onions. Int. J. Agric. Biol. 11, 463–467. doi: 10.3763/ijas.2009.0459

[B27] Graham J. H. Syversen J. P. (1984). Influence of vesicular-arbuscular mycorrhiza on the hydraulic conductivity of roots of two Citrus root stocks. New Phytol. 97, 277–284. doi: 10.1111/j.1469-8137.1984.tb04132.x

[B28] Gucwa-Przepióra E. Chmura D. Sokołowska K. (2016). AM and DSE colonization of invasive plants in urban habitat: a study of Upper Silesia (southern Poland). J. Plant Res. 129, 603–614. doi: 10.1007/s10265-016-0802-7 26894756 PMC4909803

[B29] He C. Wang W. Q. Hou J. L. (2019). Plant growth and soil microbial impacts of enhancing licorice with inoculating dark septate endophytes under drought stress. Front. Microbiol. 10. doi: 10.3389/fmicb.2019.02277 PMC6794389 31649632

[B30] Hodge A. Campbell C. D. Fitter A. H. (2001). An arbuscular mycorrhizal fungus accelerates decomposition and acquires nitrogen directly from organic material. Nature 413, 297–299. doi: 10.1038/35095041 11565029

[B31] Huang J. P. Ji M. X. Xie Y. K. Wang S. S. He Y. L. Ran J. J. (2016). Global semi-arid climate change over last 60 years. Clim. Dyn. 46, 1131–1150. doi: 10.1007/s00382-015-2636-8

[B32] Huertas V. Jiménez A. Diánez F. Chelhaoui R. Santos M. (2024). Importance of dark septate endophytes in agriculture in the face of climate change. J. Fungi 10, 329. doi: 10.3390/JOF10050329 PMC11122602 38786684

[B33] Jumpponen A. Mattson K. G. Trappe J. M. (1998). Mycorrhizal functioning of *Phialocephala fortinii* with *Pinus contorta* on glacier forefront soil: interactions with soil nitrogen and organic matter. Mycorrhiza 7, 261–265. doi: 10.1007/s005720050190 24578052

[B34] Kapoor D. Singh S. Kumar V. Romero R. Prasad R. Singh J. (2019). Antioxidant enzymes regulation in plants in reference to reactive oxygen species (ROS) and reactive nitrogen species (RNS). Plant Gene 19, 100182. doi: 10.1016/j.plgene.2019.100182

[B35] Kumar D. Singh R. Upadhyay S. K. Verma K. K. Tripathi R. M. Liu H.. (2024). Review on interactions between nanomaterials and phytohormones: Novel perspectives and opportunities for mitigating environmental challenges. Plant Sci. 340, 111964. doi: 10.1016/j.plantsci.2023.111964 38159611

[B36] Li X. He X. L. Zhou Y. Hou Y. T. Zuo Y. L. (2019). Effects of dark septate endophytes on the performance of *Hedysarum scoparium* under water deficit stress. Front. Plant Sci. 10. doi: 10.3389/fpls.2019.00903 PMC6637391 31354772

[B37] Li M. Hou L. Liu J. Yang J. Zuo Y. Zhao L.. (2021). Growth-promoting effects of dark septate endophytes on the non-mycorrhizal plant *Isatis indigotica* under different water conditions. Symbiosis 85, 291–303. doi: 10.1007/S13199-021-00813-0

[B38] Li X. Liu F. (2016). “Drought stress memory and drought stress tolerance in plants: biochemical and molecular basis,” in Drought Stress Tolerance in Plants, vol. 1 . Eds. Hossain I. M. A. Wani S. H. Bhattacharjee S. Burritt D. J. Tran L. S. P. (Springer, Berlin), 17–44. doi: 10.1007/978-3-319-28899-4_2

[B39] Li M. Ren Y. He C. Yao J. Wei M. He X. (2022a). Complementary effects of dark septate endophytes and trichoderma strains on growth and active ingredient accumulation of *Astragalus mongholicus* under drought stress. J. Fungi 8, 920–920. doi: 10.3390/JOF8090920 PMC9506129 36135646

[B40] Li M. T. Yuan C. Zhang X. H. Pang W. B. Zhang P. P. Xie R. Z.. (2022b). The transcriptional responses of ectomycorrhizal fungus, *Cenococcum geophilum*, to drought stress. J. Fungi 9, 15–15. doi: 10.3390/JOF9010015 PMC9864566 36675836

[B41] Liu N. Zhao Z. Y. Jiang X. L. Xing X. K. (2021). Review and prospect of researches on the mechanisms of mycorrhizal fungi in improving plant drought resistance. Mycosystema 214, 851–872. doi: 10.13346/j.mycosystema.200370

[B42] Lu N. Yu M. Cui M. Luo Z. J. Feng Y. Cao S.. (2016). Effects of different ectomycorrhizal fungal inoculates on the growth of *Pinus tabulaeformis* seedlings under greenhouse conditions. Forests 7, 316. doi: 10.3390/f7120316

[B43] Mandyam K. Loughin T. Jumpponen A. (2010). Isolation and morphological and metabolic characterization of common endophytes in annually burned tallgrass prairie. Mycologia 102, 813–821. doi: 10.3852/09-212 20648749

[B44] Meena K. K. Sorty A. M. Bitla U. M. Choudhary K. Gupta P. Pareek A.. (2017). Abiotic stress responses and microbe-mediated mitigation in plants: the omics strategies. Front. Plant Sci. 8. doi: 10.3389/fpls.2017.00172 PMC5299014 28232845

[B45] Pal M. Tajti J. Szalai G. Peeva V. Vegh B. Janda T. (2018). Interaction of polyamines, abscisic acid and proline under osmotic stress in the leaves of wheat plants. Sci. Rep. 8, 12839. doi: 10.1038/s41598-018-31297-6 30150658 PMC6110863

[B46] Phillips J. M. Hayman O. S. (1970). Improved procedures for clearing roots and staining parasitic and vesicular-arbuscular mycorrhizal fungi for rapid assessment of infection. Trans. Br. Mycol. Soc 55, 158–160. doi: 10.1016/S0007-1536(70)80110-3

[B47] Quiroga G. Erice G. Aroca R. Zamarreño Á.M. García-Mina J. M. Ruiz-Lozano J. M. (2020). Radial water transport in arbuscular mycorrhizal maize plants under drought stress conditions is affected by indole-acetic acid (IAA) application. J. Plant Physiol. 246-247, 153115. doi: 10.1016/j.jplph.2020.153115 31958683

[B48] Raja V. Majeed U. Kang H. Andrabi K. John R. (2017). Abiotic stress: Interplay between ROS, hormones and MAPKs. Environ. Exp. Bot. 137, 142–157. doi: 10.1016/j.envexpbot.2017.02.010

[B49] Rezaei-Chiyaneh E. Mahdavikia H. Subramanian S. Alipour H. Siddique K. H. M. Smith D. L. (2021). Co-inoculation of phosphate-olubilizing bacteria and mycorrhizal fungi: Effect on seed yield, physiological variables, and fixed oil and essential oil productivity of ajowan (*Carum copticum* L.) under water deficit. J. Soil Sci. Plant Nutr. 21, 3159–3179. doi: 10.1007/s42729-021-00596-9

[B50] Salehi-Lisar S. Y. Bakhshayeshan-Agdam H. (2016). “Drought stress in plants: causes, consequences, and tolerance,” in Drought Stress Tolerance in Plants, vol. 1 . Eds. Hossain M. Wani S. Bhattacharjee S. Burritt D. Tran L. S. (Springer, Cham), 1–16. doi: 10.1007/978-3-319-28899-4_1

[B51] Sanad M. N. Smertenko A. Garland-Campbell K. A. (2019). Differential dynamic changes of reduced trait model for analyzing the plastic response to drought phases: a case study in spring wheat. Front. Plant Sci. 10. doi: 10.3389/fpls.2019.00504 PMC6497792 31080454

[B52] Santos M. Cesanelli I. Diánez F. Sánchez-Montesinos B. Moreno-Gavira A. (2021). Advances in the role of dark septate endophytes in the plant resistance to abiotic and biotic stresses. J. Fungi 7, 939. doi: 10.3390/JOF7110939 PMC8622582 34829226

[B53] Santos S. G. D. Silva P. R. A. D. Garcia A. C. Zilli J. E. Berbara R. L. L. (2017). Dark septate endophyte decreases stress on rice plants. Braz. J. Microbiol. 48, 333–341. doi: 10.1016/j.bjm.2016.09.018 28089614 PMC5470451

[B54] Saritha M. Kumar P. Panwar N. R. Burman U. (2021). Intelligent plant-microbe interactions. Arch. Agron. Soil Sci. 68, 1002–1018. doi: 10.1080/03650340.2020.1870677

[B55] Sharma S. Verslues P. E. (2010). Mechanisms independent of abscisic acid (ABA) or proline feedback have a predominant role in transcriptional regulation of proline metabolism during low water potential and stress recovery. Plant Cell Environ. 33, 1838–1851. doi: 10.1111/j.1365-3040.2010.02188.x 20545884

[B56] Subramanian K. Santhanakrishnan P. Balasubramanian P. (2006). Responses of field grown tomato plants to arbuscular mycorrhizal fungal colonization under varying intensities of drought stress. Sci. Hortic. 107, 245–253. doi: 10.1016/j.scienta.2005.07.006

[B57] Surono S. Narisawa K. (2017). The dark septate endophytic fungus *Phialocephala fortinii* is a potential decomposer of soil organic compounds and a promoter of *Asparagus officinalis* growth. Fungal Ecol. 28, 1–10. doi: 10.1016/j.funeco.2017.04.001

[B58] Tedersoo L. Bahram M. Toots M. Diédhiou A. G. Henkel T. W. Kjøller R.. (2012). Towards global patterns in the diversity and community structure of ectomycorrhizal fungi. Mol. Ecol. 21, 4160–4170. doi: 10.1111/J.1365-294X.2012.05602.X 22568722

[B59] Tedersoo L. Brundrett M. (2017). Evolution of ectomycorrhizal symbiosis in plants. Ecol. Stud. 230, 407–467. doi: 10.1007/978-3-319-56363-3_19

[B60] Tedersoo L. Drenkhan R. Abarenkov K. Anslan S. Bahram M. Bitenieks K.. (2024). The influence of tree genus, phylogeny, and richness on the specificity, rarity, and diversity of ectomycorrhizal fungi. Environ. Microbiol. 16, e13253–e13253. doi: 10.1111/1758-2229.13253 PMC10994715 38575147

[B61] van der Heijden M. G. A. (2002). “Arbuscular mycorrhizal fungi as a determinant of plant diversity: in search of underlying mechanisms and general principles,” in Mycorrhizal Ecology. Eds. Heijden M. G. A. Sanders I. R. (Springer Berlin Heidelberg, New York, NY), 244–265.

[B62] Veresoglou S. D. Chen B. D. Rillig M. C. (2012). Arbuscular mycorrhiza and soil nitrogen cycling. Soil Biol. Biochem. 46, 53–62. doi: 10.1016/j.soilbio.2011.11.018

[B63] Vergara C. Araujo K. E. C. Urquiaga S. Santa-Catarina C. Schultz N. da Silva A. E.. (2018). Dark septate endophytic fungi increase green manure-^15^N recovery efficiency, N contents, and micronutrients in rice grains. Front. Plant Sci. 9. doi: 10.3389/fpls.2018.00613 PMC5946629 29780402

[B64] Vergara C. Araujo K. E. C. Urquiaga S. Schultz N. de Carvalho Balieiro F. Medeiros P. S.. (2017). Dark septate endophytic fungi help tomato to acquire nutrients from ground plant material. Front. Microbiol. 8. doi: 10.3389/fmicb.2017.02437 PMC5732191 29312163

[B65] Wang J. Zhang H. Gao J. Zhang Y. Liu Y. Tang M. (2021). Effects of ectomycorrhizal fungi (*Suillus variegatus*) on the growth, hydraulic function, and non-structural carbohydrates of *Pinus tabulaeformis* under drought stress. BMC Plant Biol. 21, 171. doi: 10.1186/S12870-021-02945-3 33838652 PMC8035767

[B66] Wu Q. S. Xia R. X. Zou Y. N. (2006). Reactive oxygen metabolism in mycorrhizal and non-mycorrhizal citrus (*Poncirus trifoliata*) seedlings subjected to water stress. J. Plant Physiol. 163, 1101–1110. doi: 10.1016/j.jplph.2005.09.001 17032615

[B67] Xu L. Niu X. Li X. Zheng Y. Feng H. Fu Q.. (2022). Effects of nitrogen addition and root fungal inoculation on the seedling growth and rhizosphere soil microbial community of *Pinus tabulaeformis* . Front. Microbiol. 13. doi: 10.3389/FMICB.2022.1013023 PMC9626767 36338078

[B68] Yin D. Deng X. Song R. (2016). Synergistic effects between *Suilllus luteus* and *Trichoderma virens* on growth of Korean spruce seedlings and drought resistance of Scotch pine seedlings. J. For. Res. 27, 193–201. doi: 10.1007/s11676-015-0131-z

[B69] Yin D. Song R. Qi J. Deng X. (2018). Ectomycorrhizal fungus enhances drought tolerance of *Pinus sylvestris* var. mongolica seedlings and improves soil condition. J. For. Res. 29, 1775–1788. doi: 10.1007/s11676-017-0583-4

[B70] Zhang R. Q. Tang M. Chen H. Tian Z. Q. (2011). Effects of ectomycorrhizal fungi on damping-off and induction of pathogenesis-relatedproteins in *Pinus tabulaeformis* seedlings inoculated with *Amanita vaginata* . For. Pathol. 41, 262–269. doi: 10.1111/j.1439-0329.2010.00669.x

[B71] Zhang X. Zhang J. He J. Li M. Matsushita N. Geng Q.. (2024). Physiological and transcriptome responses of *Pinus massoniana* seedlings inoculated by various ecotypes of the ectomycorrhizal fungus *Cenococcum geophilum* during the early stage of drought stress. J. Fungi 10, 71. doi: 10.3390/JOF10010071 PMC10817269 38248980

[B72] Zhang Z. (1990). Plant physiology Experiment Instruction (Beijing: Higher Education Press).

[B73] Zhao M. Hao W. Y. Ning X. Z. Hao L. F. Yan H. X. Mu Y. N.. (2020). Screening of excellent ectomycorrhizal fungi-tree for drought resistant with *Pinus sylvestris* var. *mongolica* . Bull. Botanical Res. 40, 133–140. doi: 10.7525/j.issn.1673-5102.2020.01.018

[B74] Zhou Y. Zheng Y. Li P. Xu L. Fu Q. (2024). Ectomycorrhizal fungi and dark septate endophyte inoculation improve growth and tolerance of *Pinus tabulaeformis* under cadmium stress. Pedosphere 34, 473–483. doi: 10.1016/J.PEDSPH.2023.09.003

