# Supplementary material for: Enhancing drought resistance in Pinus tabuliformis seedlings through root symbiotic fungi inoculation
Source: Front Plant Sci. 2024 Aug 20;15:1446437. doi: 10.3389/fpls.2024.1446437 (PMC11368727; doi:10.3389/fpls.2024.1446437)
Supplement: Supplementary file 1 [file DataSheet1.docx]

Supplementary Material

## Supplementary Figures

##
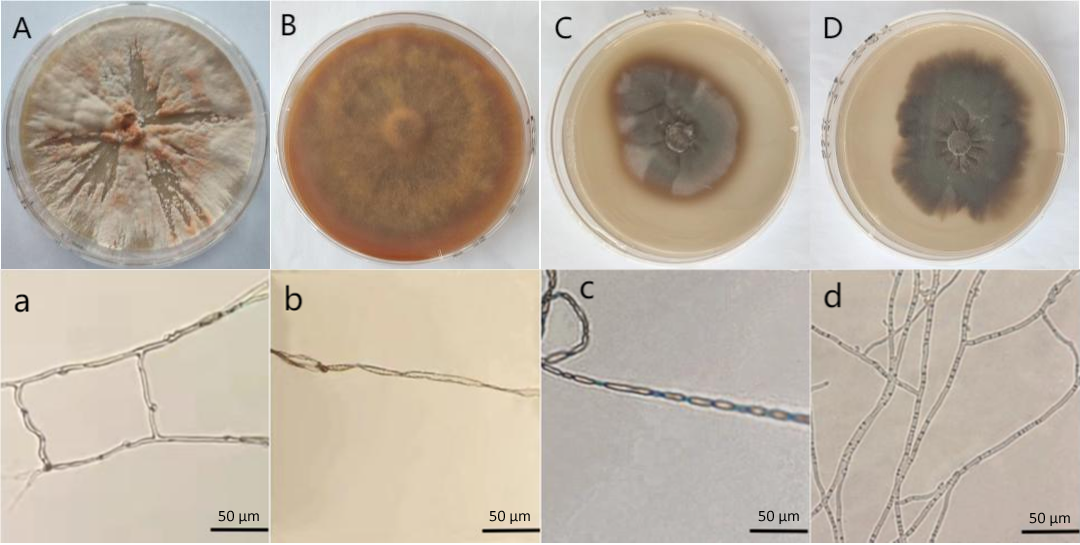


**Figure S1** Morphology of fungal colonies and mycelium grown on potato dextrose agar (PDA) medium. (**A**), colony morphology of *Sg*. (**B**), Colony morphology of *Pt*. (**C**), colony morphology of *Po*. and (**D**), colony morphology of *Ps*. (**a**), mycelial morphology of *Sg*. (**b**), mycelial morphology of *Pt*. (**c**), mycelial morphology of *Po*. and (**d**), mycelial morphology of *Ps*. Note: Scale bars = 50 μm. *Sg*: *Suillus granulatus*; *Pt*: *Pisolithus tinctorius*; *Po*: *Pleotrichocladium opacum*; *Ps*: *Pseudopyrenochaeta sp.*.

##



**Figure S2** Phylogenetic tree based on 18S rDNA gene ITS sequence analysis of fungi isolated from roots of *Pinus tabuliformis*. Sequences that were determined in the course of this study appear in bold. Fungi1: *Suillus granulatus*; Fungi2: *Pisolithus tinctorius*; Fungi3: *Pleotrichocladium opacum*; Fungi4: *Pseudopyrenochaeta sp.*.

## Supplementary Tables

**Table S1** Analysis of variance (ANOVA) of the effects of PEG stress and fungal species on the growth and physiological characteristics of root symbiotic fungi

|  | PEG stress | | Fungal species | | PEG stress × Fungal species | |
| --- | --- | --- | --- | --- | --- | --- |
|  | F | p | F | p | F | p |
| Biomass | 191.563 | < 0.001 | 1143.595 | < 0.001 | 83.098 | < 0.001 |
| SOD | 309.193 | < 0.001 | 180.371 | < 0.001 | 29.783 | < 0.001 |
| CAT | 308.236 | < 0.001 | 158.965 | < 0.001 | 52.212 | < 0.001 |
| Proline | 236.013 | < 0.001 | 878.854 | < 0.001 | 261.586 | < 0.001 |
| Soluble protein | 306.921 | < 0.001 | 445.967 | < 0.001 | 269.884 | < 0.001 |

Significant *p*-values (< 0.05) are in bold.

**Table S2** Analyses of variance (ANOVA) for the effects of drought stress and fungal inoculation on the growth and physiological parameters of *Pinus tabuliformis* seedlings.

| Indexes | Drought stress | | Fungal inoculation | | Drought stress × Fungal inoculation | |
| --- | --- | --- | --- | --- | --- | --- |
|  | F | p | F | p | F | p |
| Fungal colonization rate | 28.528 | < 0.001 | 15.032 | < 0.001 | 2.602 | 0.027 |
| Mycorrhizal growth response | 65.003 | < 0.001 | 42.628 | < 0.001 | 5.993 | < 0.001 |
| Plant height | 24.822 | < 0.001 | 21.987 | < 0.001 | 4.648 | < 0.001 |
| Shoot biomass | 7.537 | 0.002 | 54.771 | < 0.001 | 4.286 | 0.001 |
| Root biomass | 3.564 | 0.039 | 34.153 | < 0.001 | 1.505 | 0.178 |
| Total biomass | 12.609 | < 0.001 | 98.092 | < 0.001 | 5.55 | < 0.001 |
| SOD | 307.177 | < 0.001 | 101.459 | < 0.001 | 19.99 | < 0.001 |
| CAT | 60.782 | < 0.001 | 10.897 | < 0.001 | 3.82 | 0.001 |
| MDA | 1501.964 | < 0.001 | 87.568 | < 0.001 | 25.503 | < 0.001 |
| Proline | 1611.456 | < 0.001 | 305.187 | < 0.001 | 351.056 | < 0.001 |
| Soluble protein | 36.259 | < 0.001 | 10.338 | < 0.001 | 0.767 | 0.658 |
| Shoot N content | 6.726 | 0.003 | 28.721 | < 0.001 | 8.868 | < 0.001 |
| Root N content | 15.06 | < 0.001 | 8.939 | < 0.001 | 4.736 | < 0.001 |
| Shoot P content | 121.468 | < 0.001 | 57.101 | < 0.001 | 17.896 | < 0.001 |
| Root P content | 279.409 | < 0.001 | 406.345 | < 0.001 | 253.306 | < 0.001 |

Significant *p*-values (< 0.05) are in bold.

**Table S3** Effects of different concentrations of PEG-6000 (0%, 15%, 25%, and 35%) on the biomass, superoxide dismutase activity, catalase activity, proline and soluble protein of four root symbiotic fungi.

| Fungal species | PEG stress treatment | Biomass (g) | SOD  (U/g) | CAT  (U/g) | Proline (μg/g) | Soluble protein  (μg/g) |
| --- | --- | --- | --- | --- | --- | --- |
|  |  |  |  |  |  |  |
|  |  |  |  |  |  |  |
| Sg | 0% | 0.40±0.07b | 107.52±5.52c | 1.12±0.01d | 29.30±0.26b | 2.01±0.08d |
|  | 15% | 0.91±0.01a | 147.36±3.25b | 1.61±0.01b | 42.80±0.59a | 4.02±0.07a |
|  | 25% | 0.90±0.10a | 172.58±5.56a | 1.85±0.01a | 29.69±0.55b | 2.97±0.04b |
|  | 35% | 0.57±0.06b | 108.73±1.96c | 1.27±0.01c | 23.30±0.14c | 2.67±0.08c |
| Pt | 0% | 0.78±0.03b | 114.43±4.92c | 0.92±0.03c | 11.20±0.26d | 2.69±0.01a |
|  | 15% | 0.65±0.02c | 129.34±1.22b | 0.91±0.01c | 24.85±0.72a | 1.58±0.01b |
|  | 25% | 0.75±0.02b | 170.40±1.82a | 1.25±0.03b | 16.31±0.16b | 1.38±0.01c |
|  | 35% | 0.99±0.01a | 117.68±5.25bc | 1.44±0.02a | 13.41±0.20c | 1.29±0.01d |
| Po | 0% | 1.49±0.01d | 116.39±3.17d | 1.11±0.02d | 46.45±0.57b | 1.64±0.11d |
|  | 15% | 1.95±0.07c | 185.98±2.14b | 1.78±0.03b | 57.95±0.75a | 2.53±0.02c |
|  | 25% | 2.70±0.12b | 206.68±1.68a | 2.04±0.0.3a | 27.81±0.98c | 2.87±0.13b |
|  | 35% | 3.05±0.16a | 173.10±4.57c | 1.56±0.05c | 25.78±0.60c | 4.43±0.07a |
| Ps | 0% | 1.33±0.01d | 112.58±1.56b | 0.81±0.11c | 26.52±1.39c | 1.24±0.06c |
|  | 15% | 4.12±0.01a | 196.15±5.33a | 2.07±0.02a | 34.95±0.50b | 3.25±0.05b |
|  | 25% | 3.44±0.05b | 202.92±1.21a | 1.82±0.03b | 37.54±0.68b | 4.45±0.09a |
|  | 35% | 3.15±0.05c | 204.85±1.83a | 1.80±0.04b | 61.29±1.61a | 4.55±0.03a |

Data (means ± SD, n = 3) are significantly different (*p* < 0.05) if followed by different letters within the same column in the same treatment Sg: *Suillus granulatus*; Pt: *Pisolithus tinctorius*; Po: *Pleotrichocladium opacum*; Ps: *Pseudopyrenochaeta sp.*.

**Table S4** Effects of different inoculation treatments on the growth performance of *Pinus tabuliformis* seedlings under drought stress.

| Drought treatment | Inoculation treatment | Fungal colonization  rate (%) | Mycorrhizal growth response (%) | Plant  Height (cm) | Shoot biomass (g) | Total biomass (g) |
| --- | --- | --- | --- | --- | --- | --- |
|  |  |  |  |  |  |  |
|  |  |  |  |  |  |  |
| WW | CK | 0 | 0 | 4.17±0.09c | 0.27±0.01c | 0.37±0.01c |
|  | Sg | 56.67±1.92cd | 18.24±1.29b | 4.57±0.09abc | 0.32±0.01b | 0.46±0.01b |
|  | Pt | 62.22±1.11bc | 18.67±1.64b | 4.87±0.18a | 0.32±0.01b | 0.46±0.01b |
|  | Po | 52.22±4.01d | 6.08±1.41c | 4.90±0.26a | 0.28±0.01c | 0.40±0.01c |
|  | Ps | 68.89±4.01ab | 7.04±1.40c | 4.33±0.09bc | 0.27±0.01c | 0.40±0.01c |
|  | Mix | 72.22±1.11a | 31.27±0.79a | 4.70±0.06ab | 0.38±0.01a | 0.54±0.01a |
| LD | CK | 0 | 0 | 3.60±0.12c | 0.24±0.01d | 0.34±0.01d |
|  | Sg | 57.78±1.11c | 27.52±0.79b | 4.53±0.26b | 0.34±0.01b | 0.47±0.01b |
|  | Pt | 71.11±5.88ab | 30.54±1.95b | 5.33±0.24a | 0.35±0.01b | 0.49±0.01b |
|  | Po | 66.67±1.92bc | 21.74±1.60c | 4.33±0.09b | 0.29±0.01c | 0.44±0.01c |
|  | Ps | 74.44±4.01ab | 29.81±1.33b | 4.23±0.12b | 0.34±0.01b | 0.49±0.01b |
|  | Mix | 81.11±4.01a | 38.1±0.85a | 5.47±0.15a | 0.39±0.01a | 0.55±0.01a |
| SD | CK | 0 | 0 | 3.57±0.03d | 0.23±0.01c | 0.33±0.01d |
|  | Sg | 57.78±2.94ab | 19.56±1.92c | 4.27±0.12ab | 0.29±0.01b | 0.42±0.01c |
|  | Pt | 51.11±1.11bc | 22.58±4.37bc | 4.17±0.20ab | 0.30±0.03b | 0.43±0.03bc |
|  | Po | 48.89±2.94c | 19.00±2.82c | 3.63±0.09cd | 0.29±0.02b | 0.41±0.01c |
|  | Ps | 63.33±1.92a | 29.64±1.51ab | 4.03±0.09bc | 0.33±0.01ab | 0.48±0.01b |
|  | Mix | 58.89±1.11a | 35.93±1.35a | 4.60±0.21a | 0.37±0.01a | 0.52±0.01a |

Data (means ± SD, n = 3) are significantly different (*p* < 0.05) if followed by different letters within the same column in the same treatment Sg: *Suillus granulatus*; Pt: *Pisolithus tinctorius*; Po: *Pleotrichocladium opacum*; Ps: *Pseudopyrenochaeta sp.*; Mix: mixed inoculation of four root symbiotic fungi. WW: well-watered; LD: light drought; SD: severe drought.

**Table S5** Effects of different inoculation treatments on the physiological characteristics of *Pinus tabuliformis* seedlings under drought stress.

| Drought treatment | Inoculation treatment | SOD  (U/gFW) | CAT (mg/(g·min)) | MDA (μmol/g) | Pproline (μg/g) | Soluble protein (μg/g) |
| --- | --- | --- | --- | --- | --- | --- |
|  |  |  |  |  |  |  |
|  |  |  |  |  |  |  |
| WW | CK | 67.24±2.33c | 0.12±0.03a | 5.72±0.41a | 57.74±0.77c | 8.59±1.31b |
|  | Sg | 105.20±0.87ab | 0.17±0.02a | 5.84±0.14a | 65.97±7.30c | 24.59±3.67ab |
|  | Pt | 112.29±4.50ab | 0.15±0.01a | 5.57±0.20a | 65.13±0.84c | 32.56±0.73a |
|  | Po | 102.98±0.87b | 0.11±0.01a | 5.29±0.19a | 127.72±1.97b | 25.84±3.10ab |
|  | Ps | 106.91±2.99ab | 0.13±0.03a | 5.45±0.09a | 154.75±3.89a | 25.27±8.65ab |
|  | Mix | 114.10±4.92a | 0.12±0.03a | 5.47±0.12a | 67.97±0.95c | 30.84±8.16a |
| LD | CK | 91.14±2.33d | 0.18±0.02c | 16.87±0.12a | 112.12±1.73f | 21.16±3.06d |
|  | Sg | 147.35±6.08c | 0.32±0.05ab | 13.03±0.10c | 153.93±3.01d | 36.88±4.67bc |
|  | Pt | 187.02±6.96b | 0.43±0.05a | 10.90±0.07de | 201.03±2.30a | 34.48±2.36c |
|  | Po | 147.66±1.69b | 0.41±0.07a | 14.83±0.26b | 194.53±0.90b | 34.17±4.13c |
|  | Ps | 181.45±1.96b | 0.21±0.02bc | 11.47±0.31d | 184.62±2.07c | 44.52±0.70ab |
|  | Mix | 227.22±10.16a | 0.45±0.03a | 10.81±0.18e | 128.62±0.85e | 50.95±1.33a |
| SD | CK | 103.09±2.33c | 0.13±0.02c | 24.30±1.02a | 137.90±2.07e | 35.44±1.81b |
|  | Sg | 128.98±2.21b | 0.26±0.04a | 16.80±0.33b | 156.37±0.45d | 46.74±4.15ab |
|  | Pt | 137.99±2.11ab | 0.29±0.02a | 14.47±0.47cd | 198.28±3.88c | 47.18±5.59ab |
|  | Po | 110.35±6.30c | 0.22±0.02ab | 17.74±0.26b | 245.58±3.98b | 43.20±6.28b |
|  | Ps | 111.30±3.78c | 0.14±0.02bc | 16.21±0.29bc | 74.09±0.67f | 50.93±8.59ab |
|  | Mix | 147.89±4.52a | 0.21±0.04abc | 14.30±0.71d | 291.05±5.40a | 64.74±4.60a |

Data (means ± SD, n = 3) are significantly different (*p* < 0.05) if followed by different letters within the same column in the same treatment Sg: *Suillus granulatus*; Pt: *Pisolithus tinctorius*; Po: *Pleotrichocladium opacum*; Ps: *Pseudopyrenochaeta sp.*; Mix: mixed inoculation of four root symbiotic fungi. WW: well-watered; LD: light drought; SD: severe drought.

**Table S6** Effects of different inoculation treatments on nitrogen and phosphorus content in the shoots and roots of *Pinus tabuliformis* seedlings under drought stress

| Drought treatment | Inoculation treatment | Shoot nitrogen  content (%) | Root nitrogen  content (%) | Shoot phosphorus content (%) | Root phosphorus content (%) |
| --- | --- | --- | --- | --- | --- |
|  |  |  |  |  |  |
|  |  |  |  |  |  |
| WW | CK | 0.11±0.01e | 0.30±0.04a | 0.07±0.01e | 0.04±0.01c |
|  | Sg | 0.12±0.01bc | 0.33±0.01a | 0.09±0.01b | 0.06±0.01b |
|  | Pt | 0.14±0.01a | 0.28±0.02a | 0.09±0.01b | 0.06±0.01b |
|  | Po | 0.11±0.01de | 0.30±0.02a | 0.08±0.01d | 0.06±0.01b |
|  | Ps | 0.12±0.01cd | 0.27±0.01a | 0.09±0.01c | 0.06±0.01b |
|  | Mix | 0.13±0.01ab | 0.28±0.03a | 0.12±0.01a | 0.08±0.01a |
| LD | CK | 0.11±0.01d | 0.23±0.01e | 0.06±0.01c | 0.05±0.01c |
|  | Sg | 0.12±0.01c | 0.30±0.01d | 0.08±0.01b | 0.05±0.01c |
|  | Pt | 0.12±0.01c | 0.35±0.01bc | 0.10±0.01a | 0.06±0.01b |
|  | Po | 0.13±0.01c | 0.32±0.01cd | 0.10±0.01a | 0.07±0.01a |
|  | Ps | 0.14±0.01b | 0.37±0.01ab | 0.08±0.01b | 0.05±0.01c |
|  | Mix | 0.16±0.01a | 0.39±0.02a | 0.10±0.01a | 0.07±0.01a |
| SD | CK | 0.10±0.01c | 0.21±0.02c | 0.06±0.01b | 0.05±0.01c |
|  | Sg | 0.11±0.01bc | 0.29±0.01b | 0.07±0.01ab | 0.05±0.01c |
|  | Pt | 0.13±0.01a | 0.25±0.01b | 0.07±0.01ab | 0.05±0.01c |
|  | Po | 0.13±0.01a | 0.28±0.01b | 0.07±0.01ab | 0.04±0.01c |
|  | Ps | 0.13±0.01a | 0.26±0.01b | 0.07±0.01ab | 0.11±0.01b |
|  | Mix | 0.12±0.01ab | 0.33±0.01a | 0.07±0.01a | 0.23±0.01a |

Data (means ± SD, n = 3) are significantly different (*p* < 0.05) if followed by different letters within the same column in the same treatment. Sg: *Suillus granulatus*; Pt: *Pisolithus tinctorius*; Po: *Pleotrichocladium opacum*; Ps: *Pseudopyrenochaeta sp.*; Mix: mixed inoculation of four root symbiotic fungi. WW: well-watered; LD: light drought; SD: severe drought.
